# DNA Extraction from Museum Specimens of Parasitic Hymenoptera

**DOI:** 10.1371/journal.pone.0045549

**Published:** 2012-10-15

**Authors:** Jeremy C. Andersen, Nicholas J. Mills

**Affiliations:** Department of Environmental Science Policy and Management, University of California, Berkeley, California, United States of America; Natural History Museum of Denmark, Denmark

## Abstract

At the same time that molecular researchers are improving techniques to extract DNA from museum specimens, this increased demand for access to museum specimens has created tension between the need to preserve specimens for maintaining collections and morphological research and the desire to conduct molecular analyses. To address these concerns, we examined the suitability of non-invasive DNA extraction techniques on three species of parasitic Hymenoptera (Braconidae), and test the effects of body size (parasitoid species), age (time since collection), and DNA concentration from each extract on the probability of amplifying meaningful fragments of two commonly used genetic loci. We found that age was a significant factor for determining the probability of success for sequencing both 28S and COI fragments. While the size of the braconid parasitoids significantly affected the total amount of extracted DNA, neither size nor DNA concentration were significant factors for the amplification of either gene region. We also tested several primer combinations of various lengths, but were unable to amplify fragments longer than ∼150 base pairs. These short fragments of 28S and COI were however sufficient for species identification, and for the discovery of within species genetic variation.

## Introduction

Methods for extracting and analyzing DNA sequence data from specimens not immediately preserved for DNA extraction are improving at a rapid rate, as highlighted by the recent sequencing of the Neanderthal genome [1]. Among these methods, several techniques exist which allow DNA to be extracted from a specimen without conferring visible damage [2], [3], [4]. These “non-invasive” techniques are of particular interest to natural history museums as they have the potential to contribute to the value of collections, with little to no cost to the museum with regard to the number and quality of specimens held. Insects are a group where these techniques have received increasing attention, and non-invasive techniques have been used for a variety of orders, including Coleoptera, Diptera, Hemiptera, Hymenoptera, Lepidoptera, and Orthoptera, as well as several non-insect arthropods belonging to the Acarina and Aranea [2], [3], [5], [6], [7], [8]. Recent attempts have been able to amplify, through polymerase chain reaction (PCR), useable fragments of mitochondrial DNA from insect specimens collected as early as 1820 [9]. DNA extracted from museum specimens has been a useful source of information for understanding recent shifts in population structure, especially with regard to population declines in native pollinators [10], [11], in addition to having been helpful in the context of molecular based identifications [3], and the short fragments of DNA extracted from museum specimens have recently been used in Next-Generation Sequencing applications [12].

Unfortunately, due in part to the increased demand by researchers for access to museum specimens, tensions exist between the need to preserve specimens for morphological research and the desire to conduct molecular analyses [13]. Part of this tension is a result of a general lack of knowledge on behalf of both researchers and museum curators as to the likelihood of successfully extracting DNA from dried specimens, the likelihood of generating meaningful sequence data for subsequent analysis, and the post-extraction quality of museum specimens used for non-invasive techniques.

One taxon for which DNA information from museum specimens is highly desirable is the parasitic Hymenoptera, in which cryptic variation is common and correct identification is notoriously difficult - even for trained specialists [14]. In addition, parasitic Hymenoptera have been the subject of many phylogenetic and evolutionary studies [15], and are important economically, because of their value in the biological control of insect pests in agricultural, urban, and forest environments [16], [17].

In this study we examine the suitability of non-invasive DNA extraction techniques for pinned specimens of three species of parasitic Hymenoptera (Braconidae). We test the effects of body size (parasitoid species), and age (time since collection) on the total amount of DNA extracted, and the effect of these three factors on the probability of amplifying meaningful fragments of two commonly used genetic loci. We then test the utility of these amplified fragments in conjunction with previously published sequences for producing phylogenetic trees, one of the primary methods for species identification, and discovery of within-species genetic variation [18]. Finally, we make recommendations regarding the suitability of non-invasive techniques for molecular analysis of less robust museum specimens.

## Materials and Methods

### Species Examined

Specimens from three species in the family Braconidae (*Atanycolus longifemoralis* Shenefelt, *Meteorus trachynotus* Viereck, and *Trioxys pallidus* Haliday) were selected from the collection of parasitic Hymenoptera housed in the Essig Museum of Entomology at the University of California, Berkeley. Permission to work with specimens was granted by the Essig Museum, and all specimens were provided on loan. These three species are represented by a large number of specimens collected over a range of years, and by individuals that have been identified by taxonomic specialists. *A. longifemoralis* is a large (2–8 mg dry weight) ectoparasitoid of wood-boring coleopteran larvae, such as *Melanophila drummondi*, found on Douglas-fir in the west United States, and British Columbia [19], [20], [21]. We examined 15 specimens of *A. longifemoralis* collected between 1931 and 1981. *M. trachynotus* is a midsize (0.1–0.3 mg dry weight) endoparasitoid of *Choristoneura* budworms in North America [22]. We examined 6 specimens of *M. trachynotus* collected either in 1914 or 1980. We also examined three unidentified specimens in the genus *Meteorus* collected in 2009. *T. pallidus* is a small (<0.03 mg dry weight) endoparasitoid that was introduced to California and Oregon for classical biological control programs of walnut (*Chromaphis juglandicola*) and filbert (*Myzocallis coryli*) aphids respectively [23], [24], [25]. We examined 12 specimens of *T. pallidus* collected between 1959 and 1993. For all specimens, collection information is provided in Table 1.

**Table 1:** Parasitoid specimens from the Essig Museum collection used for DNA extraction, indicating age, weight, extracted DNA concentration and success of sequencing the two selected genetic loci (together with base pair length).

ID #	Location	Age (Years)	Collection Date	Weight (mg)	DNA (ng/ul)	28S (bp)	COI (bp)
*Atanycolus longifemoralis* Shenefelt							
J0075	Yosemite, CA	79	5.vi.1931	4.013	141.93	No	No
J0076	Fallen Leaf Lake, CA	70	5.vii.1940	8.145	55.04	Yes (140)	No
J0077	Fallen Leaf Lake, CA	70	5.vii.1940	3.763	44.58	Yes (140)	No
J0078	6 mi east of Chester, CA	56	14.vii.1954	5.273	113.53	Yes (140)	No
J0079	6 mi east of Chester, CA	56	14.vii.1954	5.011	66.08	No	No
J0080	6 mi east of Chester, CA	56	14.vii.1954	6.096	310.10	No	No
J0081	Hobart Mills, CA	48	29.vii.1962	5.873	26.08	Yes (140)	No
J0082	7 mi north of Truckee, CA	48	29.vii.1962	2.584	63.36	No	No
J0083	7 mi north of Truckee, CA	48	29.vii.1962	2.780	115.70	No	No
J0084	2 mi west of Brancomb, CA	34	25–27.v.1976	3.225	84.28	Yes (140)	No
J0085	2 mi west of Brancomb, CA	34	25–27.v.1976	4.236	506.16	Yes (140)	No
J0086	2 mi west of Brancomb, CA	34	25–27.v.1976	2*	74.00	Yes (140)	Yes (103)
J0087	Echo Lake, CA	29	24.vi.1981	1.588	70.32	Yes (140)	Yes (103)
J0088	Echo Lake, CA	29	24.vi.1981	1.739	113.22	Yes (140)	Yes (103)
J0089	Tahoe City, CA	29	30.ix.1981	4.425	28.10	Yes (140)	Yes (103)
*Meteorus trachynotus* Viereck							
J0103	Orono, ME	96	27.vii.1914	0.161	13.70	No	No
J0104	Orono, ME	96	27.vii.1914	0.256	152.82	No	No
J0105	Orono, ME	96	26.vii.1914	0.295	5.90	No	No
J0106	La Jara Canyon, NM	30	5.vii.1980	0.122	37.59	Yes (139)	No
J0107	La Jara Canyon, NM	30	5.vii.1980	0.256	34.21	Yes (139)	No
J0108	La Jara Canyon, NM	30	4.vii.1980	0.258	64.50	Yes (139)	No
*Meteorus* undet							
J0109	Santa Cruz, CA	1	21.5.2009	0.198	78.95	Yes (789)	Yes (658)
J0110	San Francisco, CA	1	3.vi.2009	0.274	51.05	Yes (789)	Yes (658)
J0111	San Francisco, CA	1	17.vi.2009	0.220	31.40	Yes (789)	Yes (658)
*Trioxys pallidus* Halliday							
J0090	Rancho Santa Fe, CA	51	18.viii.1959	†	11.10	Yes (155)	Yes (128)
J0092	Rancho Santa Fe, CA	51	18.viii.1959	†	9.27	No	No
J0093	U.C. Insectary, Albany, CA	47	2.v.1963	†	1.70	Yes (155)	Yes (128)
J0094	U.C. Insectary, Albany, CA	47	2.v.1963	†	8.06	Yes (155)	Yes (128)
J0095	U.C. Insectary, Albany, CA	47	2.v.1963	†	5.83	No	No
J0096	Citrus Exp. Station, Riverside, CA	33	1977	†	1.30	No	No
J0097	Citrus Exp. Station, Riverside, CA	33	1977	†	84.41	No	Yes (128)
J0098	Citrus Exp. Station, Riverside, CA	33	1977	†	11.80	No	No
J0099	U.C. Insectary, Albany, CA	17	13.vii.1993	0.006	4.80	No	No
J0100	U.C. Insectary, Albany, CA	17	13.vii.1993	0.008	13.72	Yes (155)	Yes (128)
J0101	U.C. Insectary, Albany, CA	17	13.vii.1993	0.014	11.00	Yes (155)	Yes (128)
J0102	Berkeley, CA	17	4.viii.1993	0.023	593.84	Yes (155)	Yes (128)

* Specimen weighed on mg scale.

† Specimen could not be removed from mounting pin.

### DNA Extraction Protocol

The general practice for extracting DNA from “ancient” specimens is to use a sterile laboratory – a space where no previous molecular work from the taxon of interest has been performed. However, if DNA extractions are to be routinely performed on insect specimens from museum collections, such as those housed in the Essig Museum, it is unlikely that new sterile laboratories will be available for each extraction event. Therefore, we used procedures we believed would minimize the risk of contamination. In addition to standard laboratory practices, all working spaces and instruments, including pipettes, were cleaned with a 10% bleach solution and allowed to air dry prior to extractions. DNA extraction was performed using the buffers and protocols described by Gilbert et al. [2] except as noted. Different methods were used to remove the specimens from their mounts. For specimens that were pinned directly, we first warmed the extraction buffer and then pipetted the warmed buffer over the pinned insect. After several minutes, gentle downwards pressure was applied using flamed sterilized forceps. If the parasitoid did not immediately release from the pin, the process was repeated. Some specimens of *M. trachynotus*, and all of the specimens of *T. pallidus* were glued to mounting points. For these individuals, warmed extraction buffer was used to loosen the bond between the card and the specimen. If after 30 min the parasitoid was still attached, flame-sterilized scissors were used to cut a small piece of the card with attached specimen from the rest of the mounting point to enable the specimen to be placed into the extraction buffer. For all extractions, the whole specimen was placed in a 1.5 ml eppendorf tube with 500 µl of extraction buffer. For *A. longifemoralis*, to fully submerge the specimens, multiple washes with the extraction buffer were required. Methods then followed Gilbert et al. [2]. The extracted DNA was suspended in 100 µl of DEPC nuclease free water (BioExpress), and its genomic content was quantified using a ND-1000 NanoDrop® (NanoDrop Technologies, Inc.), before being stored at −20°C.

### Remounting of Specimens

After specimens had been in 95% ethanol for at least 12 h they were removed and placed dorsally on a microscope cover slip. Enough ethanol was then added to cover the specimen, and the wings and legs were manipulated and spread prior to remounting. The ethanol was then allowed to evaporate, while the specimen was adjusted with forceps. Specimens were allowed to air dry for at least 48 h before being weighed on a Mettler-Toledo AT21 Comparator microgram balance (Mettler-Toledo International, Inc.). After measurement, individuals of *A. longifemoralis* were re-pinned. For *Meteorus* spp. and *T. pallidus*, the insects were re-glued to mounting points. Specimens were then catalogued for return to the collections at the Essig Museum of Entomology.

### DNA Amplification and Sequencing

The ability to amplify two commonly used DNA fragments, the D2 expansion region of the ribosomal gene 28S, and a fragment of the “barcoding region” of the mitochondrial gene Cytochrome Oxidase I (COI), were evaluated. For the amplification of 28S we used the forward and reverse primers, s3660 [26] and 28Sb [27], respectively, and two novel forward and reverse primers, Essig28SF2 5′ – TTG TCG GCG TGC ACT TCT C – 3′ and Essig28SR2 5′ – GAG AAG TGC ACG CCG ACA A – 3′, respectively. For the amplification of COI we used the forward and reverse primers LCO, and HCO [28], respectively, one novel forward primer BracCOIF 5′ – CAT GCW TTT RTW ATR ATT TTT TTT ATR GTW ATR CC – 3′, and three genus specific reverse primers, AtanyCOIR 5′ – CTT AAA ATT AAT AAW ATT AAT GAA GG – 3′, MeteorCOIR 5′ – TTA WAG ATA AWG GRG GRT AMA CWG TTC AHC C – 3′, and TrioxysCOIR 5′ – CAA CCC GTA CCA GCC CCT ACA TTT ATT AAA CCC C – 3′. Novel primers were designed using published sequences from congeners in GenBank as a template, and either using the software PriFi [29] or by eye.

Standard PCR protocols were followed using a BioRad Dyad programmable thermocycler (BioRad Laboratories, Inc.). PCR reactions were carried out using Amplitaq GOLD DNA polymerase and buffers (Life Technologies), with the following conditions; 2.5 µl of 10× PCR Buffer II, 1.5 µM of MgCl_2_, 0.2 µM of dNTP (Promega Corporation), 0.2 µM of each primer, 0.2 µl of Taq polymerase, 1 µl of DNA template, finally H_2_0 was added to bring the final reaction volume to 25 µl. For the amplification of 28S, all possible primer combinations were tested for all individuals, with an initial denaturing step at 94°C for 4 min was followed by thirty-five cycles of 94°C for 1 min, 52°C for 1 min, and 72°C for 1 min. This was followed by a 5 min extension step at 72°C. For the amplification of COI, genus specific reverse primers as well as the universal reverse primer “HCO” were used in combination with either the forward primer “LCO” or “BracCOIF” following the touchdown protocol presented by Hebert et al. [30]. For all primer combinations, reactions were held at 17°C, and results visualized on a 1.5% agarose gel. Sequencing of both forward and reverse fragments was performed on an Applied BioSystems 3730xl DNA Analyzer (Life Technologies) at the University of California Berkeley DNA Sequencing Facility. Sequence results were edited using Geneious Pro v. 5.5.4 [31], and Nexus files containing both sequence data, parameters for phylogenetic analyses, and tree files for each dataset can be found at TreeBase.org (accession number TB2:S12519).

### Statistical Analysis

All statistical analyses were performed using the statistical software package R v. 2.14 [32]. Differences in DNA concentration (ng/µl) between extracts from parasitoid species were assessed by analysis of covariance (ANCOVA) in the R package STATS [32] with age (in years since collection) included as a covariate. DNA concentration was log transformed to meet assumptions of normality. Backwards, stepwise model simplification was used to examine the significance of interaction terms and main effects, and after simplification, differences in DNA concentration between parasitoid species were assessed using analysis of variance (ANOVA) with Tukey's Honest-Significance test.

To analyze the probability of amplifying meaningful sequences of the two gene fragments, 28S and COI, we performed logistic regression analyses using generalized linear models (GLM), as part of the R package STATS [32], with a Bernoulli distribution (failure/success to amplify either fragment) and a logit-link function, with parasitoid species, age, and log DNA concentration as factors. Multimodel inference was performed based on Akaiki's Information Criterion corrected for small sample size (AICc) [33], [34], [35] using the R package AICcmodavg [36]. Scores were calculated for all model subsets, though the final set of retained models did not include interaction terms due to the extreme differences observed in parameter estimate standard errors (SE) [37]. Model weights were used to estimate the relative importance of each of the factors included in the models, and model averaging to provide averaged estimates and confidence intervals for each factor [38]. As age was the most important factor in the models, simplified models that included age only were used to estimate the effect of age on the probability of amplifying meaningful fragments of 28S and COI for specimens between 0 and 96 years old. These simplified models do not account for all of the variability determined by our multimodel analysis, but may be a useful first approximation in the selection of specimens prior to DNA extraction.

### Phylogenetic Analysis

One of the primary methods of analysis to resolve questions of species identification is the production of phylogenetic trees [18]. To be useful for reconstructing accurate phylogenetic relationships, however, sequence fragments must be sufficiently divergent as to differentiate individuals, whilst not being too divergent that their relationships are clouded by too much “noise.” For short fragments, this presents a particular problem, and thus quantitative analyses have been performed seeking to optimize the location, length and variability of DNA sequences [39]. To test the utility of short sequence fragments from the two gene regions, to correctly identify known and unknown specimens, as well as to reconstruct meaningful evolutionary relationships between those individuals, we used Maximum Parsimony (MP) to analyze the fragments produced in this study, with sequence data published in GenBank from either the species in question, and/or from congeners. We analyzed both gene regions separately. Alignments were generated using the sequence alignment program MUSCLE [40]. For analysis of the COI fragment, due to the high degree of sequence divergence between the three species, individual datatsets for each species (including congeners) were created, again using MUSCLE. Matrices were visualized in MacClade v. 4.08 [41], and for all analyses, datasets were truncated to correspond to the sequence fragment generated from our closest primer combinations (Essig28SF2 and Essig28SR2 for 28S; BracCOIF with either AtanyCOIR, MeteorCOIR, or TrioxysCOIR for COI), and primer regions were then excluded. MP analyses were performed using PAUP* v. 4b10 [42] for each matrix using a heuristic search algorithm with a tree-bisection-reconnection branch-swapping algorithm. For the individual analysis of the 28S dataset, gap positions were coded as a 5^th^ character state. Confidence in tree topology was estimated using 1000 bootstrap replicates.

## Results

### DNA Extraction and Sequencing

**Figure 1:**
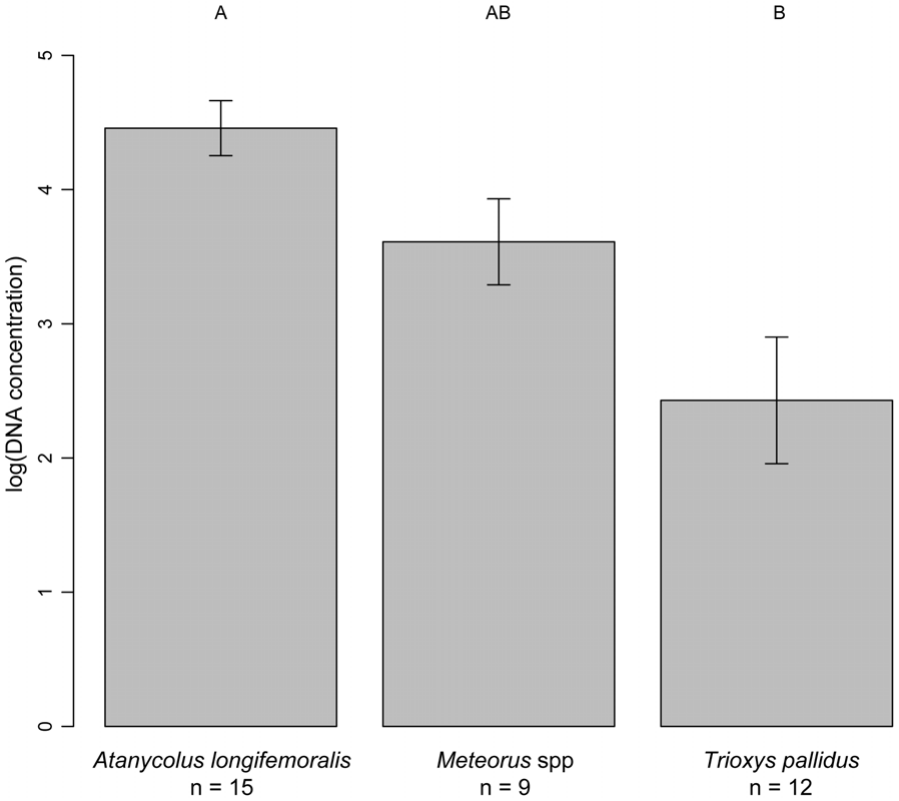
Mean DNA concentrations from three braconid species. Mean (±1 SE) DNA concentrations (ng/µl) extracted from three braconid species, as measured with a NanoDrop. Statistical differences between the species (p<0.05) are signified by a different letter above each column.

Genomic material was extracted from 15 specimens of *A. longifemoralis*, 9 specimens in the genus *Meteorus*, *and* 12 specimens of *T. pallidus*, with specimens ranging in age at time of extraction from 1 to 96 years. Results from the ANCOVA analysis showed that the total amount of genomic material (DNA concentration) differed significantly between parasitoid species (F = 10.19, df = 2,30, p<0.001), while age had no effect on DNA concentration (F = 1.73, df = 1,30, p = 0.19), and there was no interaction between age and DNA concentration (F = 1.06, df = 2, 30, p = 0.36). Post-hoc analyses found that DNA concentration differed significantly between specimens of the largest parasitoid species, *A. longifemoralis*, and the smallest parasitoid species, *T. pallidus* (p<0.001) (Figure 1).

**Figure 2:**
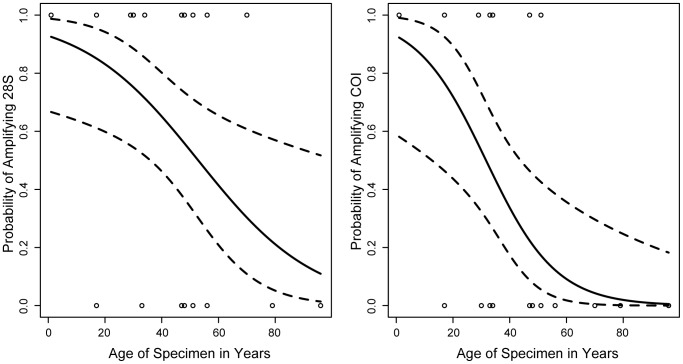
Probability of amplifying fragments of 28S and COI. The probability of successfully amplifying the 28S (left) and COI (right) gene fragments for specimens between 0 and 96 years old were estimated using the results from a logistic regression model with failure/success of amplification of each gene fragment as the response variable and age as the predictor variable. Circles represent the outcome for individual specimens, and the fitted curve from the logistic regression analysis is shown as a solid line, with associated 95% confidence intervals indicated by broken lines. For 28S the intercept equals 2.564±0.945 (t = 2.714, p = 0.01), with a slope of −0.049±0.02 (t = −2.463, p = 0.019), and for COI, the intercept equals 2.561±1.125 (t = 2.275, p = 0.023), with a slope of −0.081±0.03 (t = −2.689, p = 0.007).

Of the examined parameters, based on AIC weights from all models, the model with age alone had the largest effect on the success/failure of amplifying 28S and COI (Table 2). The sum of the Akaike weights for each model in which age appeared were 0.99 for 28S and 1.00 for COI, compared to 0.31 for 28S and 0.23 for COI for models including DNA concentration, and 0.33 for 28S and 0.3 for COI for models including parasitoid species. In addition, after model averaging, and based on weighted parameter and unconditional standard error estimates, for both 28S and COI, age was the only supported parameter based on 95% confidence intervals (Table 3). The logistic regression models using age as the only predictor variable for the amplification of 28S had an intercept of 2.564±0.945 (t = 2.714, p = 0.01) and a slope of −0.049±0.02 (t = −2.463, p = 0.019), and for the amplification of COI, an intercept of 2.561±1.125 (t = 2.275, p = 0.023) and a slope of −0.081±0.03 (t = −2.689, p = 0.007), see Figure 2.

**Table 2:** GLM model summaries for the probability of amplifying meaningful sequences of 28S and COI from three braconid parasitoid species.

Model Description	K	AIC_c_	Δ*_i_*	*w_i_*	Log-likelihood
28S∼Age	2	43.83	0	0.43	−19.73
28S∼Age, Parasitoid species	4	44.85	1.02	0.26	−17.78
28S∼Age, log(DNA)	3	45.09	1.26	0.23	−19.17
28S∼Age, log(DNA), Parasitoid species	5	47.47	3.64	0.07	−17.73
28S∼log(DNA)	2	51.53	7.7	0.01	−23.58
28S∼Parasitoid species	3	53.94	10.11	0	−23.59
28S∼log(DNA), Parasitoid species	4	56.19	12.36	0	−23.45
COI∼Age	2	38.36	0	0.53	−17
COI∼Age, Parasitoid species	4	39.98	1.62	0.24	−15.34
COI∼Age, log(DNA)	3	40.64	2.28	0.17	−16.94
COI∼Age, log(DNA), Parasitoid species	5	42.57	4.21	0.06	−15.29
COI∼Parasitoid species	3	51.91	13.55	0	−22.58
COI∼log(DNA)	2	52.45	14.09	0	−24.04
COI∼log(DNA), Parasitoid species	4	53.16	14.8	0	−21.94

Model names, descriptions, and AIC summaries for supported models examining factors contributing to the amplification of fragments of 28S and COI. K = the number of fitted parameters in the model, AIC_c_ = AIC score corrected for small sample sizes, Δ*_i_* = the difference between the AIC_c_ of the current model and that of the model with the lowest AIC_c_ score, *w_i_* = Akaike weights indicating the probability of the model being the correct model compared to all other tested models.

**Table 3:** Model averaged estimates and uncertainty for the amplification of 28S and COI.

			95% CI	
Parameter	Averaged parameter estimate	Weighted unconditional SE	Upper	Lower
**28S**				
Intercept	2.785	1.495	5.699	−0.130
Age	−0.054	0.022	**−0.011**	**−0.097**
log(DNA)	0.198	0.329	0.840	−0.444
Parasitoid species (*Meteorus*)	0.186	1.407	2.929	−2.557
Parasitoid species (*Trioxys*)	−1.740	1.035	0.278	−3.757
**COI**				
Intercept	2.840	1.470	5.707	−0.026
Age	−0.089	0.034	**−0.022**	**−0.156**
log(DNA)	−0.025	0.344	0.646	−0.695
Parasitoid species (*Meteorus*)	−1.798	1.545	1.214	−4.811
Parasitoid species (*Trioxys*)	0.756	1.097	2.895	−1.383

Model-averaged parameter estimates were calculated by averaging parameter estimates over all models in which a specific predictor was included. The new averaged parameter estimates are reported with standard errors (SE), as well as 95% confidence intervals (CI). Those parameters whose 95% CI did not include zero are highlighted in bold. Summaries for the categorical parameter Parasitoid species are reported relative to Parasitoid species (*Atanycolus*).

### Phylogenetic Utility

**Figure 3:**
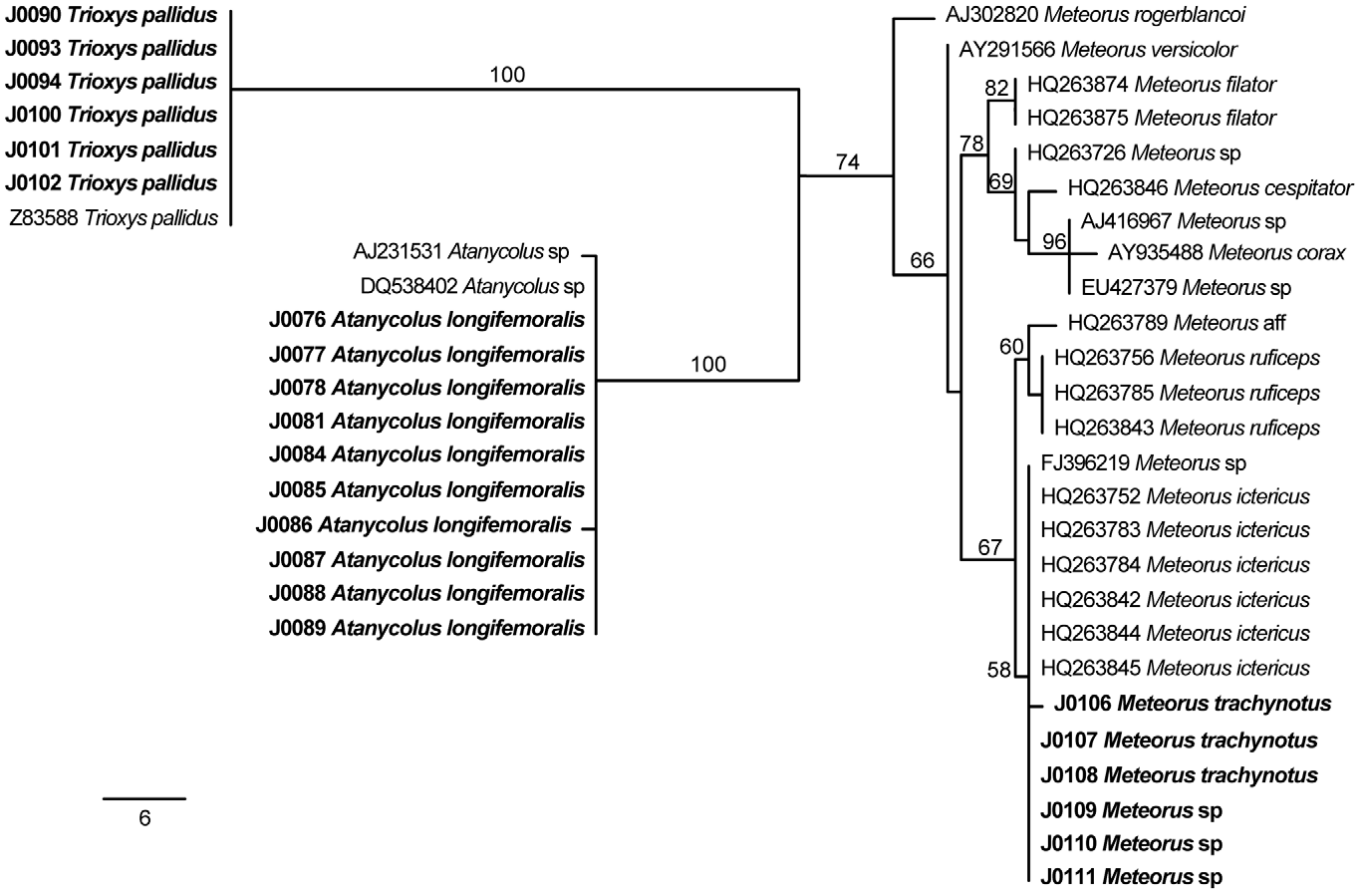
One of eight most parsimonious reconstructions of the 28S dataset. Phylogram showing of one of the most parsimonious trees from the analysis of the 28S dataset. Bootstrap support values are shown either above or next to each supported branch. Sequences generated in this study are in bold. A scale bar indicating branch-lengths is shown in the bottom left.

**Figure 4:**
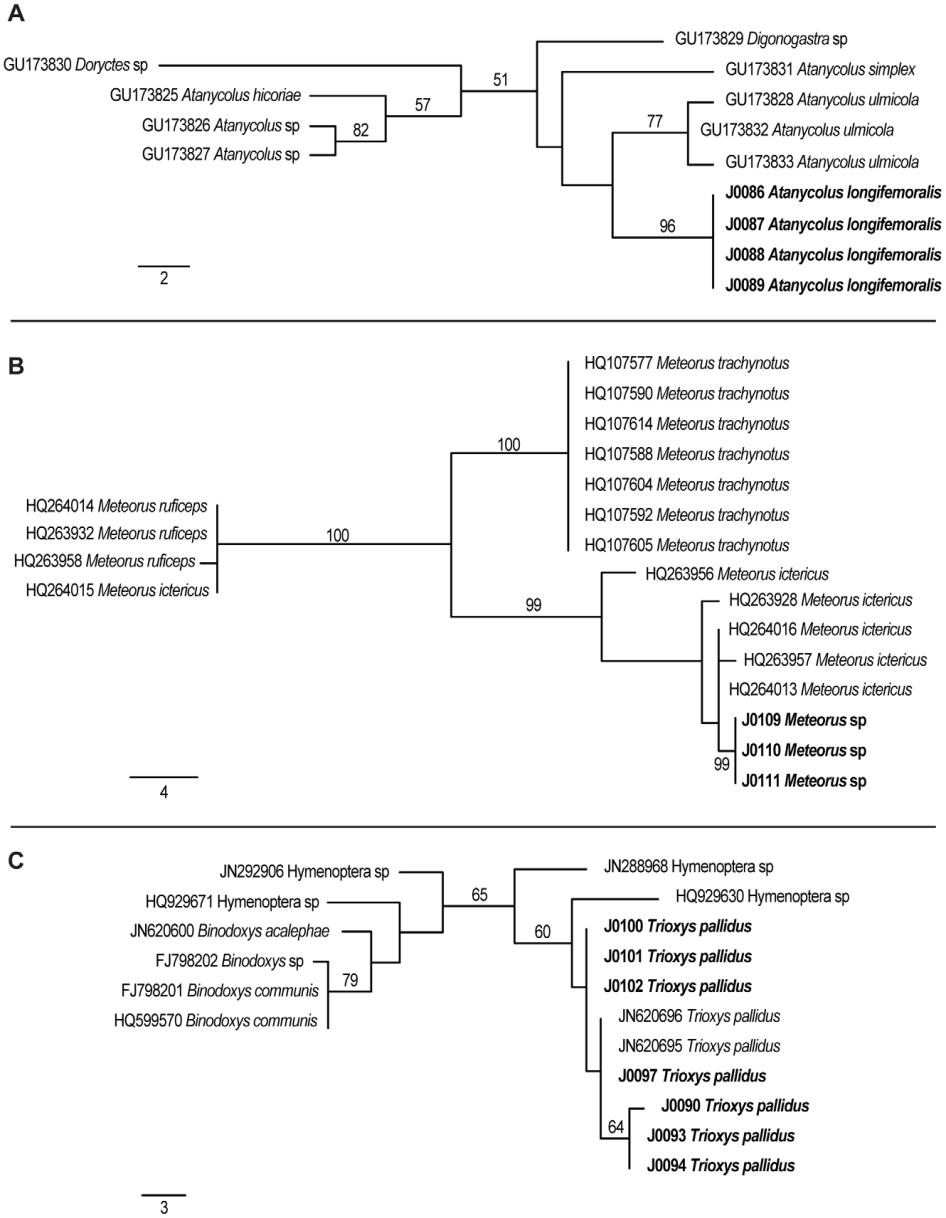
Examples of most parsimonious trees from the analyses of the COI datasets. Phylograms showing of one of the most parsimonious trees from the analysis of the COI datasets for A) *A. longifemoralis* (one of 13 MP trees), B) *Meteorus* spp., (one of 10 MP trees) and C) *T. pallidus* (one of two MP trees). Bootstrap support values are shown either above or next to each supported branch. Sequences generated in this study are in bold. For each dataset, a scale bar indicating branch-lengths is shown in the bottom left.

For the analysis of the 28S dataset, four MP trees were reconstructed (Figure 3). Sequences from all specimens formed clades with sequences from congeneric species published in GenBank with high bootstrap support (B.P.) for *A. longifemoralis* (100% B.P.) and *T. pallidus* (100% B.P.), and medium support for *Meteorus* (74% B.P.). For the analysis of the COI datasets, 13 MP trees were reconstructed for the *A. longifemoralis* dataset, 10 MP trees for the *Meteorus* spp. dataset, and 2 MP trees for the *T. pallidus* dataset (Figure 4). Relationships between *A. longifemoralis* and its closest included congener *A. ulmicola* were unsupported. Our unidentified specimens of *Meteorus* formed a highly supported clade (99% B.P.) with published sequences from *M. ictericus*, and our specimens of *T. pallidus* formed a poorly supported clade (65% B.P.) with two published sequences from *T. pallidus*, as well as two published sequences from unidentified Hymenoptera specimens.

## Discussion

### DNA Extraction and Sequencing

Recently, DNA extracted from insect specimens from museum collections has been used to illuminate questions regarding the population structure and phylogeny of a variety of insect taxa [2], [3], [4], [5], [6], [7], [8], [11], [12], [43], [44]. This study is the first to our knowledge to use these techniques with specimens of parasitic Hymenoptera, and the first that attempts to examine the effects of age, size, and DNA concentration of extracts from museum specimens on the probability of successfully sequencing meaningful fragments from those specimens. In general, we found that age had no effect on the amount of total DNA extracted from a braconid parasitoid specimen, but was a significant factor for determining the probability of success for sequencing both fragments of 28S and COI. While specimen size (represented by parasitoid species) significantly affected the total amount of extracted DNA, neither it nor DNA concentration were found to be significant factors for the amplification and sequencing of meaningful fragments of either locus based on 95% confidence intervals.

Some studies [5], [9], [10] have reported being able to amplify fragments of DNA from specimens collected more than 100 years ago. While we were able to successfully amplify and sequence short fragments of both 28S and COI from museum specimens of parasitic Hymenoptera; the oldest specimen from which we obtained 28S was 71 years (collected in 1940), and the oldest specimen from which we obtained COI was 52 years (collected in 1959). In general, we were more successful at amplifying fragments of 28S than fragments of COI, which could be due to a difference in the number of copies of these loci, or even differential rates in which these gene regions are fragmented after an organism's death, though we did not examine either of these possibilities and can only speculate with regard to their importance. Also, compared to Gilbert et al (2007) whose methods we followed, we had a slightly lower rate of success for amplifying 28S (61% compared to 78%) and a much lower rate of success for amplifying COI (38% compared to 71%). Based on our regression analysis, we found that these success rates also decreased with age, with success decreasing at a faster rate for COI than for 28S. We should note that we did not consider the effects of a specimen's temporal history, and assumed that all the specimens in this study were subject to similar storage conditions (temperature, humidity, etc.) while in the Essig Museum. Research has shown that the temporal history of a specimen can affect the success of amplification of DNA from ancient specimens [45], and researchers examining specimens from multiple natural history collections should consider the possible effects of storage history on their results.

The size of the fragments amplified in this study are similar to those reported in the majority of studies using insect specimens from museum collections [5], [8], [11], [12], [43], [44], and in particular to that observed by Rowe et al. [46], who found that the majority of the total DNA extracted from their specimens was comprised of fragments between 150 and 300 base pairs, and by Ugelvig et al. [44] who examined the length of microsatellite alleles amplified from museum specimens and found that as specimens increase in age, the length of amplifiable fragments decreases. Conversely, Tagliavia et al. [6] report being able to amplify fragments of both mitochondrial and nuclear genes of up ∼800 base pairs from specimens collected 50 years ago, and particularly for phylogenetic studies, their techniques could be of exceptional utility.

While we found no correlation between specimen age and extractable DNA concentration, and DNA concentration was not a significant factor for fragment amplification and sequencing, we caution that it may play an indirect role in the success of amplification based on the following four concerns; 1) that as a specimen ages, total DNA from the specimen may remain unchanged but become increasingly fragmented and unsuitable for PCR, 2) that as a specimen ages, total DNA from the specimen itself decreases, but over-all DNA concentrations can remain unchanged as bacteria or fungi growing in or on the specimen increase in abundance, 3) that a Nano-Drop, which cannot distinguish between single and double stranded DNA, is not the correct tool for quantifying DNA fragments from critical specimens and alternative methods which only examine double stranded DNA, or include fragment length may be more appropriate, or 4) that residual phenol from the DNA extraction process can mask the true DNA concentration, and that for older specimens these effects may be more pronounced.

### Phylogenetic Utility

The phylogenetic analysis of the 28S gene region produced clades that were well supported (Figure 4). Our analysis found no difference between specimens of *T. pallidus* at 28S, and a single base pair difference between specimens of *A. longifemoralis*. There was also a single base pair difference between one specimen of *M. trachynotus*, and the other specimens of *M. trachynotus and Meteorus* sp, as well as published sequences for *M. ictericus* and a published sequence from an unidentified *Meteorus*. The fragment of 28S, while not as variable as the fragment of COI we amplified, appeared to be useful for resolving both higher level taxonomic relationships, as well as species level differences between most of the species of *Meteorus* included in this analysis. 28S may not be sufficient however for differentiating between very closely related species (e.g. members of the same species group) as evident from the lack of differentiation between *M. ictericus* and *M. trachynotus*. The fragment of COI that we amplified was more variable than the fragment of 28S (as expected), but was amplified from fewer individuals, and in general more recent specimens (Table 1). Using the results of our phylogenetic analysis, we suspect that our unidentified specimens of *Meteorus* sp. are specimens of *M. ictericus* based on the well-supported clade they formed (100% bootstrap support) with all but one of the published sequence for *M. ictericus* by Stigenberg & Ronquist [47]. The one published sequence of *M. ictericus* which was not a member of the clade (HQ264015) was identical to several sequences from *M. ruficeps* and we expect that this represents a labeling error during the GenBank submission process. We also uncovered multiple haplotypes for *T. pallidus* within the specimens stored in the Essig Museum.

### Damage to Specimens

Though we did not quantify damage to specimens, unfortunately visible damage was observed for several of the specimens used in this study. *A. longifemoralis* has a long ovipositor, legs, and large wings, and while great care was taken to minimize damage to these structures, the ovipositor sheaths in particular were quite fragile and frequently became dislocated during the DNA extraction process. In all cases, dislocated limbs and ovipositor sheaths were glued to a mounting point on the same pin as the specimen. The major source of damage to specimens was a slight tearing of the wings that resulted from specimens becoming affixed to the glass cover slip during the 48 hr drying period after DNA extraction. All observed damage was done during specimen handling, and was not caused by the DNA extraction process directly. In general, however, specimens did appear to be lighter in color after DNA extraction, and this was most pronounced in the abdomen, though these differences were not quantified. Thus the specific method for DNA extraction used in this study may not be appropriate for specimens for which shades of color is either a distinguishing character or adds to the value of the specimen.

### Conclusions

Of the variables we examined in this study, the age of a museum specimen appears to be the most important in determining the probability of amplifying and sequencing meaningful fragments of DNA from parasitic Hymenoptera. We were able to amplify fragments of 28S from older specimens than was the case for fragments of COI. Since 28S exists at a higher copy number than COI, we suspect that as the copy number of a target DNA fragment decreases, the probability of amplifying it successfully from museum specimens will also decrease. Though the DNA fragments produced in this study were relatively short compared to those commonly used for phylogenetic or species identification applications, they were useful both for determining within species variation and for species level identification. For the reconstruction of deeper phylogenetic relationships it may be possible to create “scaffolds” of many short fragments of a target gene region in order to produce sequence data of sufficient length and diversity for analysis, to create a concatenated matrix of short fragments from two or more gene regions, or to use alternative extraction techniques which may be more effective than the methods examined here at preserving longer fragments of DNA from museum specimens [6].

## References

[1] Green RE, Krause J, Briggs AW, Maricic T, Stenzel U, et al (2011) A draft sequence of the Neandertal genome. Science 328: 710–722. 10.1126/science.1188021 PMC5100745 20448178

[2] Gilbert MTP, Moore W, Melchior L, Worobey M (2007) DNA extraction from dry museum beetles without conferring external morphological damage. PLoS ONE 2: e272. 17342206 10.1371/journal.pone.0000272 PMC1803022

[3] Rowley DL, Coddington JA, Gates MW, Norrbom AL, Ochoa RA, et al (2007) Vouchering DNA-barcoded specimens: test of a nondestructive extraction protocol for terrestrial arthropods. Mol Ecol Notes 7: 915–924.

[4] Rohland N, Hofreiter M (2007) Comparison and optimization of ancient DNA extraction. BioTechniques 42: 343–352. 17390541 10.2144/000112383

[5] Bluemel JK, King RA, Virant-Doberlet M, Symondson WOC (2011) Primers for identification of type and other archived specimens of *Aphrodes* leafhoppers (Hemiptera, Cicadellidae). Mol Ecol Resour 11: 770–774. 21457478 10.1111/j.1755-0998.2011.03008.x

[6] Tagliavia M, Massa B, Albanese I, La Farina M (2011) DNA extraction from Orthoptera museum specimens. Anal Lett 44: 1058–1062.

[7] Nagy ZT, Breman FC, Dall'Asta U (2010) DNA barcoding of museum specimens of Lymantriidae preserved in the Royal Museum for Central Africa. Entomol Rom 15: 11–16.

[8] Lis JA, Ziaja DJ, Lis P (2011) Recovery of mitochondrial DNA for systematic studies of Pentatomoidea (Hemiptera: Heteroptera): successful PCR on early 20(th) century dry museum specimens. Zootaxa 2748: 18–28.

[9] Thomsen PF, Elias S, Gilbert MTP, Haile J, Munch K, et al (2009) Non-destructive sampling of ancient insect DNA. PLoS ONE 4: e5048. 19337382 10.1371/journal.pone.0005048 PMC2660418

[10] Strange JP, Knoblett J, Griswold T (2009) DNA amplificaiton from pin-mounted bumble bees (*Bombus*) in a museum collection: effects of fragment size and specimen age on successful PCR. Apidologie 40: 134–139.

[11] Lozier JD, Cameron SA (2009) Comparative genetic analyses of historical and contemporary collections highlight contrasting demographic histories for the bumble bees *Bombus pensylvanicus* and *B. impatiens* in Illinois. Mol Ecol 18: 1875–1886. 19344350 10.1111/j.1365-294X.2009.04160.x

[12] Shokralla S, Zhou X, Janzen DH, Hallwachs W, Landry J-F, et al (2011) Pyrosequencing for mini-barcoding of fresh and old museum specimens. PLoS ONE 6: e21252. 21818256 10.1371/journal.pone.0021252 PMC3144868

[13] Mandrioli M (2008) Insect collections and DNA analyses: how to manage collections? Museum Manage Curator 23: 193–199.

[14] Noyes JS (1994) The reliability of published host-parasitoid records: a taxonomist's view. Nor J Agric Sci Suppl 16: 59–69.

[15] Dolphin K, Quicke DLJ (2001) Estimating the global species richness of an incompletely described taxon: an example using parasitoid wasps (Hymenoptera: Braconidae). Biol J Linn Soc Lond 73: 279–286.

[16] Van Driesche RG, Hoddle MS, Center T (2008) Control of Pests and Weeds by Natural Enemies. An Introduction to Biological Control. Malden, MA: Blackwell Publishing.

[17] Mills N (2000) Biological control: the need for realistic models and experimental approaches to parasitoid introductions. In: Hochberg ME, Ives AR, editors. Parasitoid Population Biology. Princeton, New Jersey: Princeton University Press. pp. 217–234.

[18] Goldstein PZ, DeSalle R (2010) Integrating DNA barcode data and taxonomic practice: Determination, discovery, and description. Bioessays 33: 135–147. 10.1002/bies.201000036 21184470

[19] Scott DW (1974) Notes on general biology of Flatheaded Fir Borer *Melanophila drummondi* Kirby reared from Ponderosa Pine (Coleoptera-Buprestidae). Pan-Pac Entomol 50: 204–205.

[20] Shenefelt RD (1943) The genus *Atanycolus* Forester in America north of Mexico. Res Stud 11: 51–163.

[21] Deyrup MA (1975) Bulletin No. 6. The Insect Community of Dead and Dying Douglas-Fir: I. The Hymenoptera. University of Washington, Seattle. 111 p.

[22] Thireau JC, Régnière J, Cloutier C (1990) Biology and morphology of immature stages of *Meteorus trachynotus* Vier. (Hymenoptera: Braconidae). Can J Zool 68: 1000–1004.

[23] van den Bosch R, Frazer BD, Davis CS, Messenger PS, Hom R (1970) *Trioxys pallidus*: an effective new walnut aphid parasite from Iran. Cal Agric 24: 8–10.

[24] van den Bosch R, Schilinger EI, Hagen KS (1962) Initial field observations in California on *Trioxys pallidus* (Haliday) a recently introduced parasite of the walnut aphid. J Econ Entomol 55: 857–862.

[25] Messing RH, AliNiazee MT (1989) Introduction and establishment of *Trioxys pallidus* [Hym.: Aphidiidae] in Oregon, U.S.A. for control of filbert aphid *Myzocallis coryli* [Hom.: Aphididae]. Entomophaga 34: 153–163.

[26] Morse GE, Normark BB (2006) A molecular phylogenetic study of armoured scale insects (Hemiptera : Diaspididae). Syst Entomol 31: 338–349.

[27] Whiting MF, Carpenter JC, Wheeler QD, Wheeler WC (1997) The Strepsiptera problem: phylogeny of the holometabolous insect orders inferred from 18S and 28S ribosomal DNA sequences and morphology. Syst Biol 46: 1–68. 11975347 10.1093/sysbio/46.1.1

[28] Folmer O, Black M, Hoeh W, Lutz R, Vrijenhoek R (1994) DNA primers for amplification of mitochondrial cytochrome c oxidase subunit I from diverse metazoan invertebrates. Mol Mar Biol and Biotechnol 3: 294–299. 7881515

[29] Fredslund J, Schauser L, Madsen L, Sandal N, Stougaard J (2005) PriFi: using a multiple alignment of related sequences to find primers for amplification of homologs. Nucleic Acids Res 33 (Web Server Issue) 16–20. 10.1093/nar/gki425 PMC1160186 15980525

[30] Hebert PDN, Cywinska A, Ball SL, deWaard JR (2003) Biological identifications through DNA barcodes. Proc R Soc Lond B Biol Sci 270: 313–321. 10.1098/rspb.2002.2218 PMC1691236 12614582

[31] Drummond A, Ashton B, Buxton S, Cheung M, Cooper A, et al. (2011) Geneious v 5.5.4, Available from http://www.geneious.com.

[32] R Development Core Team (2011) R: A Language and Environment for Statistical Computing. Vienna, Austria: R Foundation for Statistical Computing.

[33] Burnham KP, Anderson DR, Huyvaert KP (2011) AIC model selection and multimodel inference in behavioral ecology: some background, observations, and comparisons. Behav Ecol Sociobiol 65: 23–35.

[34] Grueber CE, Nakagawa S, Laws RJ, Jamieson IG (2011) Multimodel inference in ecology and evolution: challenges and solutions. J Evol Biol 24: 699–711. 21272107 10.1111/j.1420-9101.2010.02210.x

[35] Symonds MRE, Moussalli A (2011) A brief guide to model selection, multimodel inference and model averaging in behavioural ecology using Akaike's information criterion. Behav Ecol and Sociobiol 65: 13–21.

[36] Mazerolle MJ (2012) AICcmodavg: Model Selection and Multimodel Inference Based on (Q)AIC(c). http://cran.r-project.org/web/packages/AICcmodavg/index.html.

[37] Agresti A, Finlay B (2009) 14.1 Model Selection Procedures. Statistical Methods for the Social Sciences. 4th ed. Upper Saddle River, NJ: Pearson Prentice Hall. pp. 441–448.

[38] Burnham KP, Anderson DR (2002) Model Selection and Multimodal Inference: A Practical Information-theoretic Approach. New York: Springer-Verlag.

[39] Martin MJ, González-Candelas F, Sobrino F, Dopazo J (1995) A method for determining the position and size of optimal sequence regions for phylogenetic analysis. J Mol Evol 41: 1128–1138. 8587110 10.1007/BF00173194

[40] Edgar RC (2004) MUSCLE: multiple sequence alignment with high accuracy and high throughput. Nucleic Acids Res 32: 1792–1797. 15034147 10.1093/nar/gkh340 PMC390337

[41] Maddison DR, Maddison WP (2005) MacClade. 4.08 ed: Sinauer Associates.

[42] Swofford DL (2003) PAUP*. Phylogenetic Analysis Using Parsimony (*and Other Methods). 4 ed. Sunderland, Massachusetts: Sinauer Associates.

[43] Lees DC, Rougerie R, Zeller-Lukashort C, Kristensen NP (2010) DNA mini-barcodes in taxonomic assignment: a morphologically unique new homoneurous moth clade from the Indian Himalayas described in *Micropterix* (Lepidoptera, Micropterigidae). Zool Scr 39: 642–661.

[44] Ugelvig LV, Nielsen PS, Boomsma JJ, Nash DR (2011) Reconstructing eight decades of genetic variation in an isolated Danish population of the large blue butterfly *Maculinea arion* . BMC Evol Biol 11: 201. 21745368 10.1186/1471-2148-11-201 PMC3146443

[45] Smith CI, Chamberlain AT, Riley MS, Cooper A, Stringer CB, et al (2001) Not just old, but old and cold. Nature 410: 771–772. 10.1038/35071177 11298436

[46] Rowe KC, Singhal S, Macmanes MD, Ayroles JF, Morelli TL, et al (2011) Museum genomics: low-cost and high-accuracy genetic data from historical specimens. Mol Ecol Resour 11: 1082–1092. 21791033 10.1111/j.1755-0998.2011.03052.x

[47] Stigenberg J, Ronquist F (2011) Revision of the Western Palearctic Meteorini (Hymenoptera, Braconidae), with a molecular characterization of hidden Fennoscandian species diversity. Zootaxa 3084: 1–95.

